# The predictive value of maternal and neonatal inflammatory biomarkers for necrotizing enterocolitis

**DOI:** 10.1007/s00431-025-06146-0

**Published:** 2025-04-28

**Authors:** Melinda Matyas, Tamás Ilyés, Madalina Valeanu, Alexandra M Crăciun, Monica Hășmășanu, Nicoleta Grosu, Gabriela Zaharie

**Affiliations:** 1 Neonatology Department, “Iuliu Hațieganu” University of Medicine and Pharmacy, Cluj-Napoca, Romania; 2 Department of Molecular Sciences, “Iuliu Hațieganu” University of Medicine and Pharmacy, Cluj-Napoca, Romania; 3 Medical Informatics and Biostatistics Department, “Iuliu Hațieganu” University of Medicine and Pharmacy, Cluj-Napoca, Romania

**Keywords:** Preterm birth, Inflammation, Necrotizing enterocolitis, Biomarker

## Abstract

More than half of preterm births are triggered by inflammatory processes at the foeto-maternal interface. These inflammatory processes can persist after birth due to the unique vulnerabilities of preterm infants, often resulting in sustained inflammation with a role in complications such as bronchopulmonary dysplasia (BPD), necrotizing enterocolitis (NEC), intraventricular hemorrhage (IVH), and periventricular leukomalacia (PVL). The aim of this study was to evaluate the predictive value of maternal inflammatory status, assessed through biomarkers of inflammation (CRP, chorioamnionitis, preeclampsia), and neonatal inflammatory markers (CRP 1, CRP 2, PCT, IL3, MMP9) on the incidence of NEC in preterm neonates. We conducted a prospective longitudinal study in the 1st Neonatology Department, Cluj-Napoca, Romania. A total of 82 preterm newborns (gestational age < 34 weeks + 6 days) were enrolled in the study. Interleukin-3 (IL3) and matrix metalloproteinase-9 (MMP9) levels were measured using the ELISA technique. Additionally, C-reactive protein (CRP) and procalcitonin (PCT) were measured in the first days of life. The correlation between these inflammatory markers and the incidence of NEC was analyzed. Furthermore, we examined the role of maternal inflammation and chorioamnionitis in relation to NEC incidence. Out of the 82 neonates enrolled, 20 developed NEC. Neonates who developed NEC had higher IL3 levels at birth. A significant positive correlation was found between maternal CRP levels and neonatal IL3 levels (*r* = 0.541, *p* < 0.001). In the NEC group, maternal CRP levels were also elevated compared to those in neonates who did not develop NEC.

*Conclusion*: Neonatal inflammation is associated with an increased incidence of NEC. Prenatal inflammatory conditions appear to trigger a persistent inflammatory process in preterm neonates, raising the risk of NEC.

**Table:** 

**What is Known:** *• NEC is a multifactorial disease of preterm newborn with multiple maternal and neonatal risk factors.*	
**What is New:** *• Evaluate the probability of NEC at preterm neonates of mothers with ongoing inflammatory conditions during pregnancy.*

**Supplementary Information:**

The online version contains supplementary material available at 10.1007/s00431-025-06146-0.

## Introduction

Necrotizing enterocolitis (NEC) is a condition predominantly affecting premature newborns, with an incidence of 3–5% and a high mortality rate ranging from 15 to 30%. It is the most common inflammatory gastrointestinal disease associated with prematurity. The pathogenesis of NEC is multifactorial, involving prematurity, inflammation, ischemia, oxidative stress, formula feeding, and alterations in the intestinal microbiome [1, 2].

The increased permeability of the intestinal barrier plays a crucial role in the development of NEC, as it facilitates bacterial translocation and the formation of intestinal lesions. It is believed that maternal inflammatory processes can significantly promote the onset of NEC in the newborn, potentially exacerbating other pathological factors. The symptoms of NEC can, in part and especially in the initial stages of the disease, mimic those of sepsis, making differential diagnosis challenging. However, as the disease progresses, NEC presents with more specific symptoms, including marked abdominal distension, bile stasis, and rectal bleeding [3–5].

Due to the unclear etiology of NEC, preventative measures targeting the disease’s pathogenic mechanisms are currently limited. Despite progress in recent years, advancements in NEC diagnosis and treatment have been slow, likely due to its complex and poorly understood pathophysiological mechanisms [6, 7]. While not all preterm neonates exposed to similar perinatal conditions develop NEC, those who do are at risk of long-term complications such as short bowel syndrome and impaired neurological development through the gut-brain axis [7–10].

Identifying biomarkers that can predict NEC in preterm infants would be valuable in managing those at higher risk. There is growing interest in markers of intestinal injury, particularly those associated with alterations in the intestinal barrier and mucosal permeability [11–13].

Several inflammatory markers, including cytokines, have been investigated for their potential to predict NEC at its early stages. These biomarkers are associated with both sepsis and NEC, in relation to the common occurrence of intestinal barrier dysfunction in both conditions [14–18].

Interleukins (IL) 1, 3, 6, and 8, tumor necrosis factor alpha (TNFα), platelet activating factor (PAF), and the expression of toll-like receptor 4 (TLR4) on the cell surface have been studied for their diagnostic value in NEC. However, the inflammatory mediators are non-specific and form part of the systemic inflammatory cascade [14–18].

IL3 modulates the expression of IL1 and the chemokines CCL2, CCL3, CCL7, and CCL12 in macrophages, increasing the peritoneal macrophage phagocytic activity. IL3 supports the survival, proliferation, differentiation, polarization, or the recruitment of immune and non-immune cells in infection and inflammatory diseases. In case of intestinal inflammation, IL3 has a beneficial role at the onset of the disease by promoting the recruitment of splenic neutrophils with high microbicidal capability into the colon [13, 17]. This link between IL3 and intestinal inflammation recommends this biomarker as a good candidate for study in relation to NEC in preterm neonates; the fact that IL3 has been less studied in this group also recommends it.

The purpose of the study is to evaluate the probability that preterm neonates of mothers with ongoing inflammatory conditions during pregnancy, assessed by measuring conventional inflammatory markers such as CRP, chorioamnionitis, and preeclampsia, develop NEC after birth. The diagnosis of NEC was established based on Bell’s criteria.

## Materials and methods

### Study design

We conducted a prospective longitudinal study in the Neonatology Department of 1 st Obstetrics Clinic, Cluj-Napoca. In the study group, we enrolled 82 preterm newborns with a gestational age < 34 weeks, admitted between May 2022 and December 2022. Inclusion criteria were as follows: preterm neonates whose mothers provided informed consent for blood sample collection, the absence of visible congenital malformations, and no chromosomal abnormalities diagnosed antenatally.

We measured the interleukin 3(IL3) and matrix – metalloproteinase (MMP 9) in all neonates of the study group from blood samples collected at birth. In parallel, we followed the evolution of CRP and PCT determined on the first day of life in the group of premature infants enrolled in the study. These determinations are carried out in a standard way in all premature infants hospitalized in our service for the evaluation of their inflammatory status.

Data regarding maternal inflammatory status were collected, including CRP values, histopathological examination of the placenta for chorioamnionitis, and the presence of preeclampsia.

The study was approved by the ethics committee of the University of Medicine and Pharmacy Cluj-Napoca. Also, written informed consents were obtained from the parents of all the infants who participated in this study.

### Assessed parameters

We measured the IL3 and MMP9 by ELISA technique from blood samples obtained at birth. The blood samples were collected into plain, separation gel, paediatric-size vacuum tubes at birth. After 30 min at room temperature, the samples were centrifuged at 3000* g* for 10 min. The separated serum was aliquoted into sterile 1.5 ml microcentrifuge tubes and stored at – 80 °C until analysis. IL 3 and MMP9 concentrations were assayed using commercial Enzyme Linked Immunosorbent Assay (ELISA) kits (Elabscience Bionovation Inc., USA) following the manufacturer’s protocols. After processing, the plates were read by an ELISA plate reader (Tecan Sunrise®, Tecan Trading AG, Switzerland); intra- and inter-assay coefficients of variation were under 10%.

The first value of CRP was measured in the first 12 h of life, and the second value after 24 h. The PCT value of the first day of life was analyzed in the study. PCT values were interpreted according to age-adapted reference ranges to account for the physiological elevation observed in the first 48–72 h of life. These are part of the current laboratory determinations, applied in our service for the evaluation and monitoring of the inflammatory syndrome in premature newborns.

For CRP determination, we used the immunoturbidimetry method with a clinical chemistry analyser (Berckman Coulter, AU 680, USA).

For the PCT value, the chemiluminescence technique was used and applied with Dix 10.

Maternal inflammatory status was evaluated by measuring CRP, using the same technique as for premature newborns.

The diagnosis of chorioamnionitis was made by the histopathological analysis of the placenta. The diagnosis of preeclampsia was established according to its definition—maternal hypertension occurring after the gestational age of 20 weeks, accompanied by proteinuria, > 300 mg in a 24-h urine collection, or a protein/creatinine ratio of > 0.3 in a single voided urine [2].

### Clinical features

The diagnosis of NEC was established based on Bell’s criteria. The type of enteral feeding at the time of its initiation was recorded for each patient in the studied group. The type of enteral feeding was followed through the prism of the protective factor against NEC of feeding with mother’s milk.

We analyzed the correlation of the inflammatory markers with NEC incidence in the study group. We also followed up the maternal inflammation and chorioamnionitis role on NEC incidence in the study group.

### Statistical analysis

The SPSS 25.0 program (SPSS Inc, Chicago, USA) was used for statistical analysis and data description. The normality of the distribution of the quantitative data was checked with the Kolmogorov–Smirnov test. The accepted error threshold was *α* = 0.05. The arithmetic mean ± standard deviation was used to describe normally distributed continuous quantitative data, and the median (quartile 1-quartile 3) was used for those that did not have a Gaussian distribution. To compare the means of the corresponding quantitative variables of two independent groups, Student’s test (*t*-test) was used if the variables were normally distributed. The nonparametric Mann–Whitney and Kruskal–Wallis tests were used to compare the means of two independent groups, in which the variables had an abnormal distribution. Chi-square or Fisher exact tests were used to compare qualitative variables. To determine the diagnostic value of some parameters, ROC (receiver operating characteristic) curves were constructed and compared. The ROC curve illustrates the graphical relationship between Sn and Sp for certain possible threshold (cutoff) values. The optimal values from the point of view of the reliability of the analyzed parameters are considered cutoff points. For each variable considered, sensitivity is represented on the abscissa, and 1-specificity (the rate of false-positive tests) on the ordinate. A variable is considered satisfactory as a diagnostic criterion if the area under the curve AUC (area under the curve) is greater than 0.8 (80%). The closer the AUC value is to 1 (AUC = 1 corresponds to the situation where the ROC curve reaches the upper-left corner of the graph), the better the diagnostic accuracy of the considered variable.

## Results

The characteristics of the study group are presented in Table 1.

**Table 1:** Characteristics of study group (maternal and neonatal)

Maternal characteristic	Study group (*n* = 82)	NEC = yes (*n* = 20)	NEC = no (*n* = 62)	*p*-value
Preeclampsia (yes)	13 (15.9%)	4 (20%)	9 (9.7%)	0.725
Chorioamnionitis (yes)	22 (26.8%)	7 (35%)	15 (24%)	0.343
Maternal CRP (mg/dl)	0.78 (0.44–1.32)	1.88 (0.69–3.35)	0.79 (0.43–1.30)	0.044
Mode of delivery				
Vaginal delivery	23 (28.1%)			
Caesarean section	59 (71.9%)			
Newborn characteristics				
Birth weight (g)	1425.65 ± 553.19	1120.00 ± 518.64	1579.90 ± 535.29	0.001
Gestational age (weeks)	29.89 ± 2.77	28.50 (25.00–30.00)	31.00 (29.50–32.00)	< 0.001
Apgar score at 1 min	6.64 ± 2.01	6.50 (4.75–7.25)	8.00 (6.00–8.00)	0.03
Apgar score at 5 min	7.8 ± 1.57	8.00 (6.00–8.25)	8.00 (8.00–9.00)	0.04
PCT (ng/ml)	2.35 (0.32–14.4)	8.56 (0.36–11.55)	0.69 (0.25–9.88)	0.19
CRP1 (mg/dl)	0.05 (0.02–0.27)	0.07 (0.02–0.17)	0.03 (0.02–0.16)	0.81
CRP2 (mg/dl)	0.24 (0.08–1.14)	0.27 (0.08–0.48)	0.22 (0.06–0.84)	0.99
MMP9 (ng/ml)	93 (41–180.5)	158.5 (60.75–212.5)	94.5 (44.25–189.75)	0.337
IL3 (pg/ml)	193.95 (106.68–277.15)	320.63 (114.78–366.91)	204.78 (108.35–263.67)	0.165
Necrotizing enterocolitis	20 (24.4%)			
Presence of vascular catheters	82 (100%)			
NEC staging				
1 A		1 (5%)		
1B		6 (30%)		
2 A		9 (45%)		
2B		2 (10%)		
3B		2 (10%)		

The group of premature neonates enrolled in the study had an average birth weight of 1425.65 ± 553.19* g*, and the gestational age was 29.89 ± 2.77. Of the 82 premature newborns enrolled, 20 newborns developed NEC.

The IL3 value was 193.95 pg/ml (106.68–277.15) and that of MMP9 was 93 ng/ml (41–180.5). Both inflammatory markers were measured from venous blood samples collected at birth from all newborns enrolled in the study.

We followed the correlation between the occurrence of NEC in the newborns from the study and various maternal conditions (Table 1). For this purpose, we analyzed the role of chorioamnionitis, maternal preeclampsia, and maternal inflammation as risk factors for NEC.

The data analysis revealed a significant correlation between the antenatal maternal CRP rise and the occurrence of NEC in premature newborns from the studied group (*p* = 0.044), but Se = 0.824 (95% CI 0.61–0.95).

We did not find a significant correlation between the occurrence of NEC and maternal chorioamnionitis or preeclampsia (*p* > 0.05). In the study group, chorioamnionitis was present in 22 cases, and preeclampsia was present in 13 mothers. Only four prematures from mothers with preeclampsia were diagnosed with NEC (Table 1).

While the presence of histologically confirmed chorioamnionitis did not significantly increase NEC risk in our cohort, neonates who developed NEC and were exposed to chorioamnionitis had markedly higher IL3 levels compared with those with NEC without chorioamnionitis (355.46 pg/ml vs 107.13 pg/ml, *p* = 0.063); this is also true of MMP9 levels (191.5 ng/ml vs 108.67 ng/ml in those with NEC and no chorioamnionitis). Although the difference in IL3 levels is not statistically relevant (likely due to the small sample size), it suggests a potential relationship between maternal chorioamnionitis, neonatal inflammatory response, and NEC development (Table 2).

**Table 2:** Characteristics of neonates with NEC based on the presence of chorioamnionitis

Characteristics (NEC = yes)	Chorioamnionitis = no (*n* = 13)	Chorioamnionitis = yes (*n* = 7)	*p*-value
Maternal CRP	1.26 ± 1.15	2.55 ± 2.31	0.143
PCT (ng/ml)	0.38 (0.31–4.81)	5.95 (0.43–24.6)	0.837
CRP1 (mg/dl)	0.02 (0.02–1.1)	0.06 (0.02–0.12)	0.669
CRP2 (mg/dl)	0.11 (0.1–0.7)	0.11 (0.05–0.79)	0.967
IL3 (pg/ml)	107.13 (101.59–122.42)	355.46 (320.63–592.39)	0.063
MMP9 (ng/ml)	108.67 ± 91.88	191.5 ± 124.61	0.483

Chorioamnionitis determines an inflammatory intrauterine environment with an effect on the fetus and on premature newborns. Through this, we analyzed the correlation of chorioamnionitis with perinatal inflammation in the newborn. We found a significant correlation between the presence of chorioamnionitis and the IL3 value of preterms (*p* = 0.001) (Table 3). We found no correlation of maternal preeclampsia with NEC (Table 1).

**Table 3:** Chorioamnionitis and neonatal inflammatory markers in the entire study group

Inflammatory marker	Chorioamnionitis = yes	Chorioamnionitis = no	*p*-value
MMP9 (ng/ml)	156 (102–256)	87 (42.5–181)	0.181
IL3 (pg/ml)	334.47 (219.22–376.44)	183.68 (106.23–258.28)	0.001

Regarding the enteral nutrition in newborns with NEC, it was initiated with breast milk in 10 cases, 9 cases received breast milk in association with formula, and in only one case, enteral feeding was carried out with preterm formula. The type of feeding had no influence on the NEC incidence (*p* = 0.922).

**Fig. 1:**
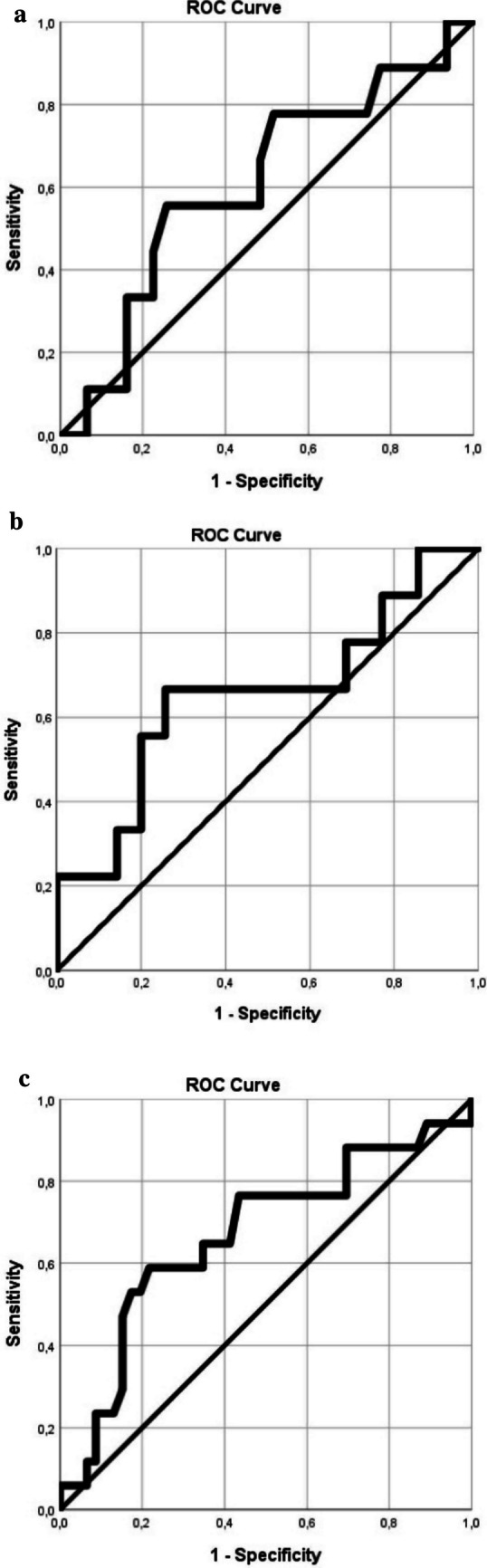
**A** ROC curve IL3; sensitivity 0.67; specificity 0.74. **B** ROC curve MMP9; sensitivity 0.22; specificity 0.83. **C** ROC curve maternal CRP; sensitivity 0.59; specificity 0.78

The sensitivity and specificity of IL3, MMP9, and CRP mother as biomarkers were analyzed using the ROC curve to confirm their ability to discriminate NEC from non-NEC. The ROC curve demonstrated by IL3 obtained an area under the curve of 0.654 (*p* > 0.05), with a cutoff value of 288.48 to discriminate NEC from non-NEC (Fig. 1A). Additionally, MMP9 obtained an area under the curve of 0.608 (*p* > 0.05), with a cutoff value of 155, and CRP-mother obtained an area under the curve of 0.667 (*p* = 0.044), with a cutoff value of 1.31 (Fig. 1B). IL3 had a sensitivity of 0.67 and a specificity of 0.74, while MMP9 had a sensitivity of 0.22 and a specificity of 0.83, and for maternal CRP, we had a sensitivity of 0.59 and specificity of 0.78 (Fig. 1C). Although our IL3 results did not reach statistical significance as standalone predictors, the relatively high sensitivity (0.67) suggests potential as part of a multi-biomarker approach to assessing the risk of NEC.

MMP9 showed much higher values in those who presented NEC 320.63 (114.78–366.91) vs 204.78 (108.35–263.67), without being statistically significant (*p* = 0.33).

## Discussion

Necrotizing enterocolitis (NEC) is a serious complication of prematurity, often associated with long-term consequences and increased mortality. This study aimed to analyze the risk of NEC development in premature infants exposed to inflammatory syndromes, by examining the role of inflammatory cytokines detected postnatally, as well as the influence of maternal inflammation on NEC risk. Cytokines play a key modulatory role in intestinal inflammation and can contribute to intestinal injury. Among these, interleukins (IL1, IL3, IL6, IL8) are frequently studied to assess inflammation in newborns. Although these interleukins are not specific to intestinal inflammation, their association with other markers may help establish an early diagnosis of NEC [19–21].

In this study, the value of IL3 was assessed in premature infants, but no statistically significant difference was found between those who developed NEC and those who did not (*p* = 0.165). However, maternal chorioamnionitis and elevated maternal C-reactive protein (CRP) were associated with a higher neonatal inflammatory status. Newborns of mothers with chorioamnionitis had significantly higher IL3 levels compared to those whose mothers did not have chorioamnionitis (*p* = 0.044).

While we did not find a direct significant association between chorioamnionitis and NEC incidence, our findings suggest that the inflammatory pathway might still be relevant. The absence of a direct relationship between chorioamnionitis and NEC, despite the relationship between chorioamnionitis and elevated IL3 levels, highlights the complex multifactorial nature of NEC pathogenesis. It is possible that chorioamnionitis creates a pro-inflammatory environment that predisposes one to NEC, but additional factors are necessary for NEC to develop.

Fetal exposure to intrauterine inflammation can lead to a sustained postnatal inflammatory response, increasing the risk of sepsis or NEC in newborns. Previous studies have described an inflammatory phenotype in these infants that increases their risk of complications [12, 22, 23]. In the studied cohort, biomarkers supported this pro-inflammatory status, with higher IL3 levels suggesting a state of sustained inflammation in newborns of mothers with chorioamnionitis, as described by Humberg et al. [24]. Approximately half of premature births are triggered by inflammation, leading to premature labor or premature rupture of membranes, often associated with or without chorioamnionitis.

Pan et al. demonstrated in their study on preterm pigs that prenatal inflammation alters the expression of genes related to both innate and adaptive immunity in the neonatal intestine, with marked immune cell infiltration observed at birth. In premature infants, pro-inflammatory cytokines like IL1, IL3, IL6, IL8, tumor necrosis factor (TNF)-α, and other inflammation-related proteins, such as CRP and intercellular adhesion molecules (ICAM- 1), are often overexpressed. The immune system’s insufficient regulation of these inflammatory markers may contribute to the development of NEC [18, 25, 26].

Evennett et al. found that CRP, while sensitive to NEC, lacks specificity and typically rises 12–24 h after NEC onset. However, it is still used routinely in neonatal care to assess treatment response. In this study, maternal CRP levels were predictive of neonatal inflammation and NEC risk. The study found CRP to have a specificity of 98% for neonatal inflammation and 30% for maternal CRP, with maternal CRP sensitivity at 82%. These findings align with other studies highlighting the high sensitivity of CRP in premature infants with NEC [27].

PCT (procalcitonin) was found to have a sensitivity of 47% and a specificity of 58% in this study. PCT, produced by thyroid parafollicular cells, regulates calcium homeostasis and rises significantly during sepsis but less so in mild NEC cases. Turner et al. found similar results. In this study, PCT levels correlated significantly with IL3 in premature infants (*p* < 0.001). The presence of elevated PCT and IL3 from birth indicates an ongoing inflammatory state, increasing the risk of NEC [15].

MMP9, a chemokine, demonstrated a specificity of 83% but a sensitivity of only 22%. Other chemokines, including MMP- 10, CCL20, and CXCL1, have also been studied in diagnosing NEC and differentiating it from neonatal sepsis. Dong et al. found that combining multiple biomarkers (IL- 8, IL- 24, CCL20) improved NEC diagnosis and sepsis differentiation [12].

Diagnosing NEC, particularly in its early stages, is challenging because symptoms often overlap with sepsis. Biomarkers like those mentioned may not be NEC-specific, but combining several of them could improve early detection. Multiple studies have evaluated the role of inflammatory biomarkers in NEC, but few have investigated the link between perinatal inflammation and NEC development in preterm infants [4, 20, 25, 28, 29].

Cetinkaya et al. analyzed SAA, PCT, and CRP in 152 preterm infants with NEC and found PCT to have the highest specificity (98%) and PPV (97%) but the lowest sensitivity (92%) [14]. Elfarargy et al. reported increased fecal calprotectin and serum levels of CRP, PCT, and ENA- 78 in the NEC group [11]. Dong et al. also found the combination of IL- 8, IL- 24, and CCL20 to be highly valuable in identifying preterm infants at high risk of NEC [12].

These studies support the idea that using multiple biomarkers could facilitate earlier NEC detection. Developing an inflammatory model with predictive value for NEC diagnosis and implementing early treatment strategies may reduce the incidence and severity of NEC in premature infants. Given the association of severe NEC with increased mortality and long-term complications, identifying high-risk groups could improve outcomes. A limitation of our study is the relatively small sample size of 82 preterms, with only 20 developing NEC. This may explain why some relationships, such as the association between IL3 levels in NEC neonates with and without chorioamnionitis exposure, approached but did not reach statistical significance (*p* = 0.063). We believe that, with a larger sample size, more definitive associations might be established. The correlation between maternal and neonatal inflammatory factors remains a key strength of this study. Further research is needed to explore inflammatory factors before and after NEC onset.

## Supplementary Information

Below is the link to the electronic supplementary material.

Supplementary file1 (DOCX 58 KB)

## Data Availability

No datasets were generated or analysed during the current study.
