# Supplementary material for: The predictive value of maternal and neonatal inflammatory biomarkers for necrotizing enterocolitis
Source: Eur J Pediatr. 2025 Apr 28;184(5):316. doi: 10.1007/s00431-025-06146-0 (PMC12037420; doi:10.1007/s00431-025-06146-0)
Supplement: Supplementary file 1 — Supplementary file1 (DOCX 58 KB) [file 431_2025_6146_MOESM1_ESM.docx]

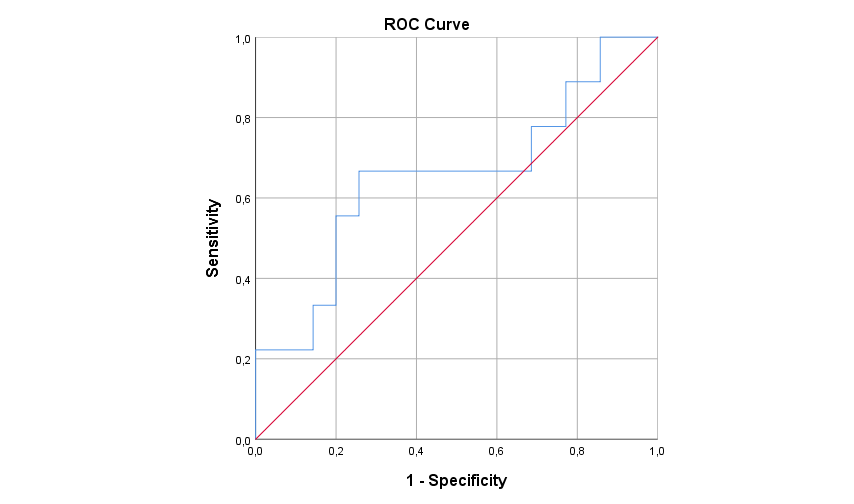


Figure 1.ROC curve IL-3; sensitivity 0.67; specificity 0.74


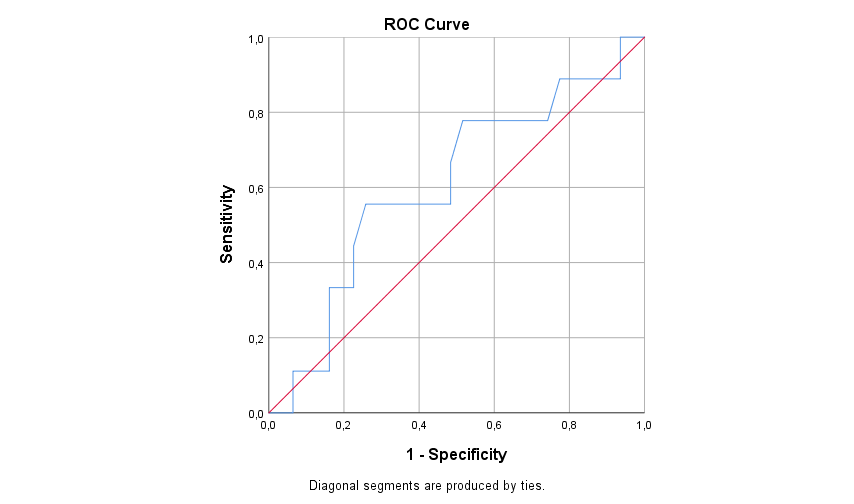


Figure 2.ROC curve MMP-9; sensitivity 0.22 ; specificity of 0.83


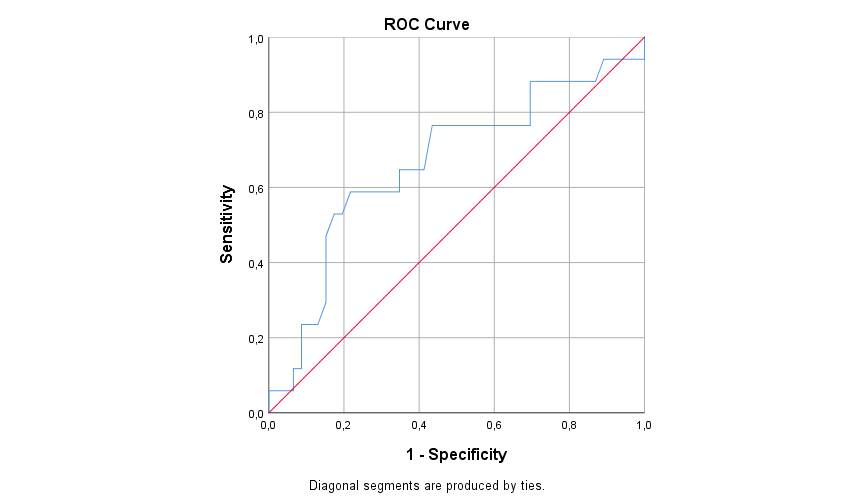


Figure 3.ROC curve maternal CRP; sensitivity 0.59 ; specificity of 0.78
